# Unilateral acute idiopathic maculopathy: angiography, optical coherence tomography and microperimetry findings

**DOI:** 10.1007/s12348-010-0014-6

**Published:** 2010-11-24

**Authors:** Miguel A. de la Fuente, Rubén Cuadrado

**Affiliations:** 1 Fundación Jiménez Díaz, Avenida Reyes Católicos 3, 28040 Madrid, Spain; 2 Optometrist, Instituto de Oftalmobiología Aplicada (IOBA), University of Valladolid, Ramón y Cajal 7, 47005 Valladolid, Spain

**Keywords:** Acute idiopathic maculopathy

## Abstract

Unilateral acute idiopathic maculopathy (UAIM) is an uncommon inflammatory disease of the retinal pigment epithelium (RPE) that affects young adults. The variability of clinical features of UAIM makes the diagnosis cumbersome. We report on a 25-year-old man with sudden loss of visual acuity (VA) and a central scotoma in his right eye. Fluorescein angiography localised the lesion in the RPE. Microperimetry revealed a central scotoma extending beyond the lesion margins with complete recovery of retinal sensitivity over weeks. Optical coherence tomography at presentation showed a thickened RPE. We are unaware of previous reports of UAIM studied by microperimetry and could find no reference to it in a computerised search using MEDLINE.

## Introduction

Unilateral acute idiopathic maculopathy (UAIM) is a rare macular disease described in 1991 by Yannuzzi et al. They reported on patients with sudden and severe unilateral visual loss, often following a flulike illness. The reduced vision was due to a greyish thickening of the RPE in the macular area, associated with an overlying exudative neurosensory retinal detachment and sometimes few intraretinal haemorrhages, papillitis and/or vitreous cells [1, 2]. The natural course of UAIM is a spontaneous recovery over a period of several weeks to months [1, 2]. The cause is still unknown, though there are reports pointing to a viral aetiology [3].

We describe the fluorescein angiography (FFA; TRC-50IX, Topcon, Tokio, Japan), optical coherence tomography (OCT; Stratus OCT 3,000, Carl Zeiss Meditec, Dublin, CA, USA) and automatic fundus-related microperimetry (MP1, Nidek Technologies, Padua, Italy) findings on a patient with UAIM in the acute stage.

## Case report

**Fig. 1:**
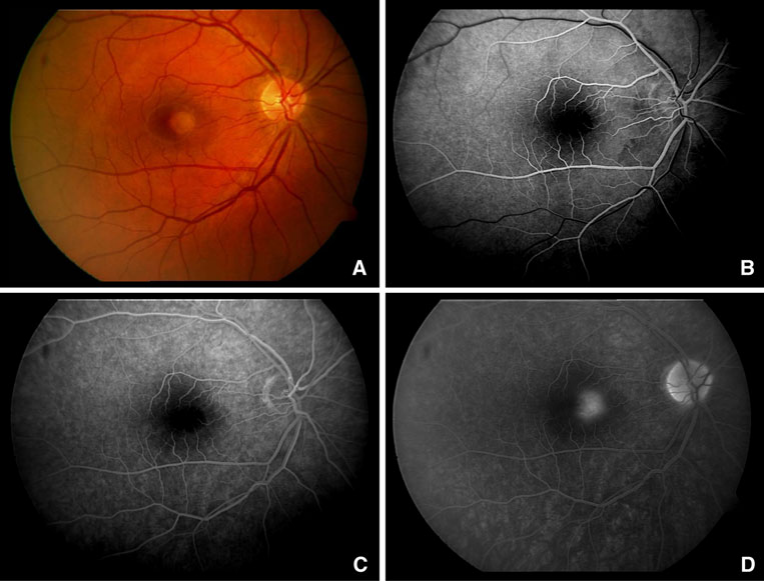
**a** Colour photograph showing a greyish lesion at the fovea. **b**, **c** Arterial and venous phases depicting the hypofluorescent lesion. **d** Late recirculation times showing hyperfluorescence

**Fig. 2:**
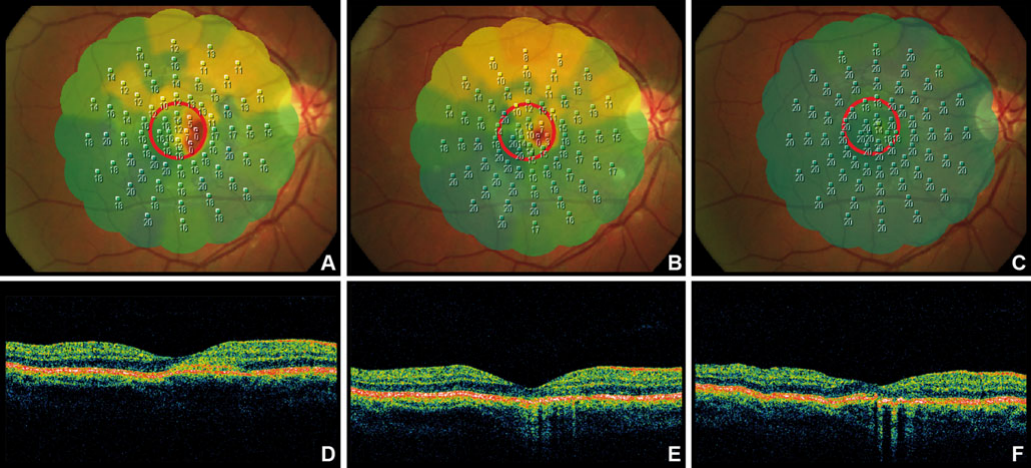
Microperimetry and OCT (horizontal scans through the fovea) at different stages of evolution. **a** Microperimetry depicting the size and depth of the scotoma at presentation. **b** Five days after therapy, the size and depth of the scotoma have diminished. **c** At 3 months, normal retinal sensitivity. **d** OCT at presentation, showing the thickened RPE protruding into the IS/OS junction. **e** Five days after therapy, hyper-reflective choroidal lines reflecting photoreceptor disruption and RPE atrophy. **f** At 3 months, hyporeflective streaks in the choroid reflecting further RPE derangement with pigment migration into the inner retina and improvement of IS/OS junction

A 25-year-old man presented to casualty with sudden loss of vision and a central scotoma in his right eye (RE). Ocular and medical histories were unremarkable. Visual acuity (VA) was 20/20 in RE and 20/16 in left eye (LE). Biomicroscopic examination showed normal anterior segments. Fundus examination of RE revealed a greyish lesion at the level of the RPE affecting the fovea. There were no cells in the vitreous. The LE was normal. Five days after the onset of symptoms, VA in RE had dropped to 20/25, and the central scotoma had enlarged. Fundus examination revealed the lesion being enlarged, threatening the foveal centre (Fig. 1a). The FFA showed a foveal hypofluorescent lesion in the early and mid-phases becoming hyperfluorescent in the late recirculation times (Fig. 1b–d). OCT depicted as a hyper-reflective RPE lesion protruding into the inner/outer segment junction (Fig. 2d). Microperimetry probed a central scotoma with marked loss of retinal sensitivity extending far beyond the lesion margins (Fig. 2a). In view of the loss of VA, the lesion growth and the foveal location, a course of systemic prednisone, starting with a dose of 1 mg/kg/daily (80 mg), was prescribed. The prednisone was then waned by 20 mg weekly.

**Fig. 3:**
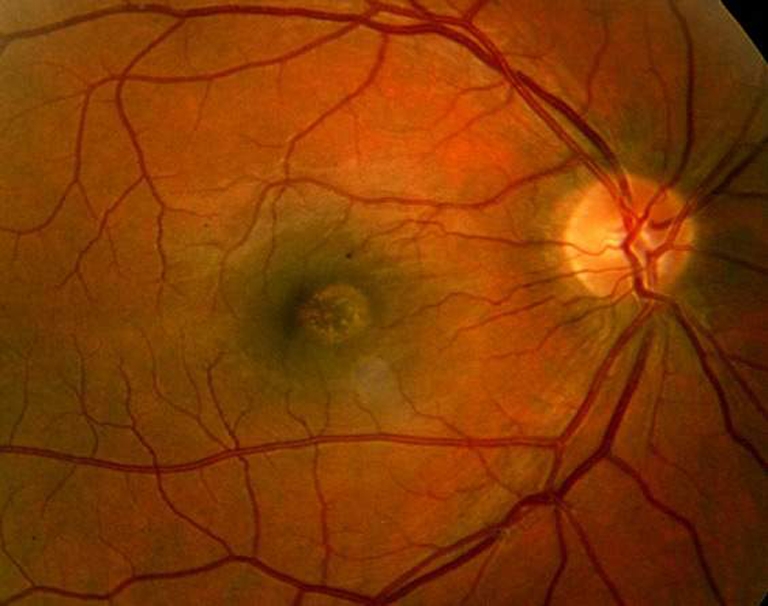
Colour picture at 2 years showing the honeycomb appearance

Five days after initiation of therapy, VA in the RE had improved to 20/20. Microperimetry revealed mild recovery of foveal sensitivity (Fig. 2b), and OCT showed a reduction in retinal thickness with hyper-reflective choroidal lines reflecting photoreceptor disruption and RPE atrophy (Fig. 2e). Three months later, VA was 20/16 in both eyes. Microperimetry was within normal values (Fig. 2c), and OCT remained with the hyper-reflective RPE specks causing hyporeflective streaks in the choroid (Fig. 2f). At 2 years follow-up, VA and retinal sensitivity remained normal, but there was a depigmented RPE lesion with focal hyperplasia with a honeycomb appearance (Fig. 3).

## Discussion

UAIM is a rare macular disease described by Yannuzzi et al. on 1991. They reported on nine young patients with unilateral profound visual loss, macular serous detachment and a central scotoma on Amsler chart testing. The same group and others expanded the clinical spectrum on the disease adding the presence of papillitis and bilaterality. The natural course was a rapid, spontaneous improvement in most patients, occurring from weeks to months [1, 2].

We report on a young patient with similar characteristics of those reported by Yannuzzi et al. but lacking the prodromal illness, a much better visual acuity and without the serous detachment.

The good VA of our patient was due to the very early stage of the disease, to the absence of serous macular detachment and to the slight eccentricity of the RPE lesion (Fig. 1a). FFA showed a hypofluorescent lesion on the early phases that became hyperfluorescent in the late recirculation times (Fig. 2b–d). We interpreted the hypofluorescence as the result of RPE oedema and the late hyperfluorescence as the breakdown of the outer blood–retinal barrier, in keeping with an active inflammatory lesion at RPE level [1]. OCT revealed either a thin layer of hyper-reflective material or oedematous RPE protruding into the inner–outer segment junction (Fig. 2d), as reported by others [3, 4].

OCT retinal thickness overlying the lesion at presentation was 305 microns, coming down to 273 microns at 3 months as the inflammation subsided. These could well be that the hyper-reflective material has been reabsorbed or the RPE oedema had settled, leaving an area of RPE derangement with pigment migration into the inner retina causing hyporeflective streaks in the choroid. There was some improvement of the IS/0S junction, corresponding with the improved vision and microperimetry (Fig. 2f). Microperimetry reflected the presence of a scotoma with deep loss of retinal sensitivity overlying the lesion (0 dB) with reduction of retinal sensitivity beyond the lesion margins (Fig. 2a) with a total recovery of sensitivity (20 dB) at 3 months (Fig. 2c), finding not previously reported. Recently, Lam et al. reported on a transient reduction of EOG amplitude in the acute stage of a patient with UAIM, suggesting a more widespread dysfunction of the RPE, in agreement with our microperimetry findings [5].

## Conclusion

In UAIM, a course of systemic steroids could hasten the resolution of inflammation and recovery of retinal sensitivity. Microperimetry and OCT seem good clinical tools to characterise and follow-up the evolution of the disease.
